# Direct Membrane Binding by Bacterial Actin MreB

**DOI:** 10.1016/j.molcel.2011.07.008

**Published:** 2011-08-05

**Authors:** Jeanne Salje, Fusinita van den Ent, Piet de Boer, Jan Löwe

**Affiliations:** 1 MRC Laboratory of Molecular Biology, Hills Road, Cambridge CB2 0QH, UK; 2 Department of Molecular Biology and Microbiology, Case Western Reserve University, Cleveland, OH 44106-4960, USA

## Abstract

Bacterial actin MreB is one of the key components of the bacterial cytoskeleton. It assembles into short filaments that lie just underneath the membrane and organize the cell wall synthesis machinery. Here we show that MreB from both *T. maritima* and *E. coli* binds directly to cell membranes. This function is essential for cell shape determination in *E. coli* and is proposed to be a general property of many, if not all, MreBs. We demonstrate that membrane binding is mediated by a membrane insertion loop in TmMreB and by an N-terminal amphipathic helix in EcMreB and show that purified TmMreB assembles into double filaments on a membrane surface that can induce curvature. This, the first example of a membrane-binding actin filament, prompts a fundamental rethink of the structure and dynamics of MreB filaments within cells.

## Highlights

► Bacterial actin MreB binds to cell membranes in a nucleotide-independent manner ► MreB assembles into double protofilaments on the surface of a lipid membrane ► Membrane binding occurs through an amphipathic helix or a membrane insertion loop ► Membrane binding by MreB is essential for its function in *E. coli*

## Introduction

MreB is the bacterial actin homolog (Jones et al., 2001; van den Ent et al., 2001). It is present in most nonspherical cells, and while many produce only a single form of MreB (*E. coli*, *C. crescentus*), others produce two or more MreB-like proteins (*T. maritima*, *B. subtilis*). MreB is essential for cell shape formation, and depletion through genetic knockouts or specific drug treatment results in the transformation into spherical cells that eventually die under usual growth conditions (Bendezú and de Boer, 2008; Lee et al., 2003; Levin et al., 1992; Wachi et al., 1987). MreB interacts directly or indirectly with several proteins, which include its operon partners MreC and MreD, the cell-shape-determining protein RodZ, cell wall synthesis components (PBPs), and even RNA polymerase (Kawai et al., 2009; Kruse et al., 2006; van den Ent et al., 2006, 2010; Vats et al., 2009; White et al., 2010).

The majority of structural and biochemical research on MreB has focused on one of the two MreBs from the thermophilic archaeum *T. maritima* (TmMreB), as it has proved difficult to purify functional MreBs from most other organisms. TmMreB assembles into filaments in the presence of ATP or GTP, and these can assemble into lateral sheets in vitro (Popp et al., 2010; van den Ent et al., 2001). The longitudinal contacts formed in TmMreB filaments are seen in the crystal structure (van den Ent et al., 2001) and closely resemble those in F-actin.

MreB filaments appear to form long spirals along the length of rod-shaped cells (Carballido-López and Errington, 2003; Figge et al., 2004; Gitai et al., 2004; Jones et al., 2001; Slovak et al., 2005; Vats and Rothfield, 2007), but two recent reports suggest that in *Bacillus subtilis* these are actually composed of short, dynamic filaments that are driven by progression of the cell wall synthesis machinery (Domínguez-Escobar et al., 2011; Garner et al., 2011). The assertion that cellular MreB filaments may not exceed 200 nm in length is supported by a recent electron tomography study that systematically searched and did not find long filaments in frozen cells (Swulius et al., 2011).

Here we show that MreBs from both *T. maritima* and *E. coli* interact directly with membranes and that this is mediated by a membrane insertion loop in TmMreB and an N-terminal amphipathic helix in EcMreB. We show that TmMreB assembles into filament doublets on a membrane surface, and that these can induce negative curvature in purified vesicles. We show that the amphipathic helix of EcMreB is both necessary and sufficient to confer membrane-binding activity, and finally demonstrate that this membrane-binding activity of EcMreB is essential for the function of MreB in cell shape determination.

## Results

### TmMreB Directly Binds and Distorts Lipid Membranes

**Figure 1:**
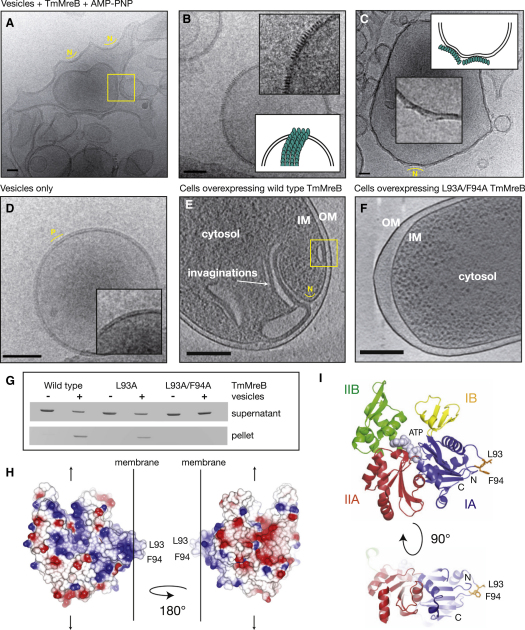
TmMreB Binds and Distorts Lipid Membranes as Shown by Electron Cryomicroscopy (A–C) Vesicles mixed with purified, untagged *Thermotoga maritima* (Tm) MreB protein (pFE349) in the presence of AMP-PNP, showing regular protein structures and gross morphological distortions. Schematic insets indicate how TmMreB (cyan) is thought to act on the bilayer. Scale bars, 50 nm. (D) Negative control showing vesicle only. Scale bar, 50 nm. (E and F) Section through a 3D electron cryotomography reconstruction of an *E. coli* cell containing high levels of wild-type, untagged TmMreB (pFE309) (E) or untagged L93A/F94A TmMreB (pJS101) (F). See also Movie S1. Protein expression levels are roughly equal as shown by whole-cell SDS-PAGE analysis in Figure S1B. Inner membrane (IM), outer membrane (OM), cytosol, and membrane invaginations are indicated. Scale bar, 250 nm. Yellow boxes indicate areas where two TmMreB surfaces interact (see also Movie S2), and yellow lines highlight regions of negative (N) and positive (P) curvature. (G) Vesicle-pelleting assay showing that purified, his-tagged TmMreB (pFE52) directly binds to membranes and that a single (MreB_L93A, pJS104) and double mutation (MreB_L93A/F94A, pJS105) in the membrane insertion loop results in partial and complete loss of membrane binding, respectively. (H) Schematic showing the known structure of TmMreB and its predicted interaction with the membrane. The residues responsible for membrane binding, L93 and F94, are highlighted, and arrows show the direction of polymerization. The protein surface is colored according to charge, with positive regions colored in blue and negative regions in red. (I) The crystal structure of TmMreB colored by domain. Domains IA, IB, IIA, and IIB are labeled, and the N and C termini are shown. Nucleotide (ATP) is shown in gray, and the residues involved in membrane binding are indicated.

Purified, nontagged TmMreB was found to bind and distort lipid vesicles as observed by electron cryomicroscopy. Vesicles alone were spherical, and a lipid bilayer could clearly be observed (Figure 1D). Once TmMreB was added, the lipid vesicles became grossly distorted and formed large clusters of protein-lipid assemblies (Figures 1A–1C). Regular structures could be made out lying close to the membrane (Figure 1B and inset), and these are interpreted as small sheets of TmMreB filaments viewed along the filament (Figure 1B, schematic inset). The structures were evenly spaced, as would be expected for sheets of MreB filaments, which have been shown previously (Popp et al., 2010; van den Ent et al., 2001). TmMreB was found to sometimes induce negative curvature (Figures 1A and 1C and inset), and this would match the negative curvature on the inside surface of the cell membrane, although the radii differ significantly.

Membrane binding and distortion by TmMreB were further confirmed when TmMreB was overexpressed to high levels in *E. coli* and cells were imaged using 3D electron cryotomography. Large membrane invaginations and internal membrane-bound structures that appeared to form by sheets of TmMreB filaments binding along the surface of the membranes could be observed in all cells (Figure 1E, see Movie S1 available online). Two sheets of membrane-bound TmMreB were frequently associated with one another along the opposite, non-membrane-binding surface in both vesicle clusters and in cells (Movie S2). The significance of this observation is not clear at present and could be caused by the high protein levels used.

Membrane binding by TmMreB is not dependent on nucleotide binding or hydrolysis. Lipid pelleting assays, which were used to confirm membrane binding by TmMreB in vitro (Figure 1G), were performed without addition of any nucleotide, and the absence of protein-bound nucleotide was confirmed by reverse-phase chromatography (Figure S1A).

### TmMreB Binds Membranes via a Hydrophobic Membrane Insertion Loop

Inspection of the structure of TmMreB revealed a small loop in domain IA that is not present in the structural homologs actin or ParM and that contains two hydrophobic residues, leucine and phenylalanine (L93, F94, Figures 1H and 1I). This loop is positioned close to the N terminus, which is composed of a further two hydrophobic residues, the start methionine and a leucine (M1, L2). We reasoned that this loop might be responsible for the membrane-binding activity of TmMreB, as it is suitably extended from the body of TmMreB and is positioned on the surface that would enable longitudinal polymerisation of TmMreB along the lipid bilayer. We constructed and purified single and double mutants in this putative membrane insertion loop (L93A and L93A/F94A) and used a vesicle-pelleting assay to test for membrane binding (Figure 1G). Compared with wild-type, we observed a partial (L93A) and complete (L93A/F94A) loss of membrane-binding activity, suggesting that this loop is indeed required for interaction with the membrane. In order to confirm this, we overexpressed L93A/F94A TmMreB in *E. coli* cells and imaged rapidly frozen cells using cryo-electron tomography. In contrast to the cells containing wild-type TmMreB, there was no evidence of any membrane invaginations (Figure 1F), and cells appeared rod shaped when observed by light microscopy (data not shown).

### TmMreB Assembles into Sheets and Double Filaments on a Membrane Surface

**Figure 2:**
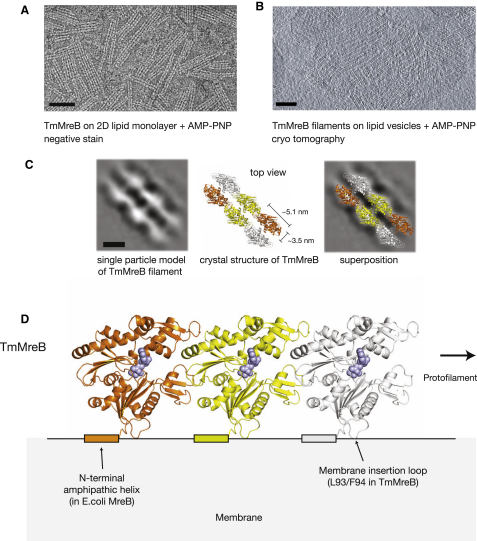
TmMreB Assembles into Double Filaments and Sheets on a Lipid Membrane Surface (A) Negative-stain electron micrograph of untagged, wild-type TmMreB (pFE349) filaments assembled on a lipid monolayer in the presence of AMP-PNP. Scale bar, 50 nm. (B) Digital section through a 3D cryo-electron tomography reconstruction of TmMreB filaments assembled on lipid vesicles in the presence of AMP-PNP. Here the membrane is composed of a bilayer rather than a monolayer. Scale bar, 50 nm. (C) (Left) Single particle reconstruction of the TmMreB double filament based on negative stain images similar to that shown in (A). Scale bar, 5 nm. The crystal structure of TmMreB protofilaments is shown to scale (middle) and docked into the single particle reconstruction (right). (D) Model for the interaction of an MreB filament with the membrane. The membrane insertion loop required for TmMreB is shown, as well as the N-terminal amphipathic helix from EcMreB.

In order to analyze the structure and organization of membrane-bound TmMreB filaments, we used negative stain electron microscopy to image TmMreB filaments that were assembled on a lipid monolayer (Figure 2A). For this, a lipid monolayer was formed on the hydrophobic surface of a carbon-coated electron microscopy grid, and this was used to attract TmMreB filaments onto the surface of the monolayer from a drop of buffer containing a very low protein concentration. Negative controls showed that in the absence of either nucleotide or lipids no filaments were observed and that the L93A/F94A membrane-binding mutant did not assemble on the monolayer (data not shown). Polymerization assays, analyzed by negative stain EM, show that the L93A/F94A mutant still polymerizes (data not shown).

The TmMreB filament structures observed on the lipid monolayer resembled some of those observed previously in the absence of any lipid (Esue et al., 2005; Popp et al., 2010; van den Ent et al., 2001). We found that TmMreB assembled into double filaments and sheets, and single protofilaments were never observed. Similar double filaments were also formed on a lipid bilayer, as shown by cryotomography (Figure 2B and Movie S2). We performed single-particle analysis on isolated double filaments (Figure 2C), and the resulting structure could easily be docked with two copies of the atomic structure of the protofilament found in the TmMreB crystals (Figure 2C). The filaments in the TmMreB crystals were composed of isolated protofilaments and therefore the lateral interaction of TmMreB protofilaments was not known. The TmMreB protofilament is characterized by a flat and a contoured surface, and our reconstruction clearly shows that the double filament is paired along the flat surface with the contoured surfaces facing out. There is a small internal density within the filament, and this is neatly matched by two protruding helices in the crystal structure. It is not possible to deduce the orientation of the filaments relative to the plane of the bilayer from this 2D reconstruction, i.e., the two filaments might be parallel or antiparallel. Taken together, TmMreB protofilaments are anchored into the membrane by a membrane insertion loop thereby orienting the protofilament along the membrane surface (Figure 2D).

### MreBs from Gram-Negative Bacteria Carry an Additional N-Terminal Region that Forms a Predicted Amphipathic Helix

**Figure 3:**
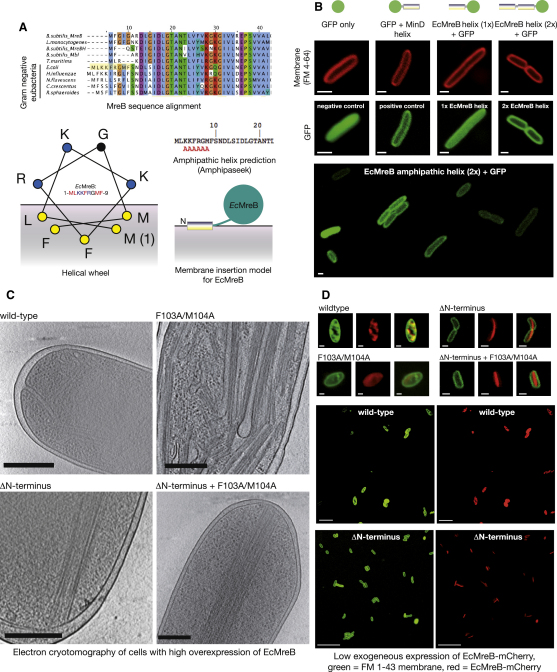
EcMreB Has a Small N-Terminal Amphipathic Helix that Is Necessary and Sufficient to Confer Membrane-Binding Activity (A) (Top) Multiple sequence alignment of MreBs. Sequences from Gram-negative organisms are highlighted and carry an additional N-terminal sequence. (Left) Helical wheel showing the view along the first nine residues of EcMreB. Hydrophobic residues are clustered on one side to form a membrane binding surface. Residues are colored by properties: hydrophobic, yellow; basic, blue; uncharged, black. (Right) Results of amphipathic helix prediction on EcMreB from AMPHIPASEEK software (http://npsa-pbil.ibcp.fr/cgi-bin/npsa_automat.pl?page=/NPSA/npsa_amphipaseek.html). Red “A”s indicate predicted amphipathic helical regions. Below is the membrane insertion model for EcMreB showing the N-terminal amphipathic helix. (B) Confocal microscopy images showing the localization pattern of GFP alone (pRSET/EmGFP) or with an additional amphipathic helix from MinD (pJS110) or one or two copies of the amphipathic helix from EcMreB (pFE356 and pJS111, respectively). Red, FM4-64 membrane stain; green, GFP. Scale bar, 1 μM. (C) Digital sections through 3D electron cryotomography reconstructions of *E. coli* cells in which EcMreB (WT or mutant) has been expressed to high levels (WT EcMreB pFE57, double mutant F103A/M104A pJS107, pJS108, ΔN terminus/F103A/M104A PJS109). Protein expression levels are roughly as shown by whole-cell SDS-PAGE analysis in Figure S3C. Scale bar, 250 nm. (D) Confocal microscopy images showing wild-type *E. coli* cells containing low-level overexpression of wild-type and mutant EcMreB-mCherry^SW^. Wild-type EcMreB-mcherry^SW^ pFE363, EcMreB-ΔN F103A/M104A-mcherry^SW^ pFE364, EcMreB-F103A/M104A-mcherry^SW^ pFE365, EcMreB-ΔN mcherry^SW^ pFE366. Green, FM1-43 membrane stain; red, mCherry. Scale bar, 1 μM.

Multiple sequence alignments of MreBs revealed that they cluster into two groups, one with and one without a short (∼7–9 residue) additional N-terminal region, and that those with the additional region were all from Gram-negative bacteria (Figure 3A). We used the amphipathic helix prediction software AMPHIPASEEK (Sapay et al., 2006) and found that all those additional regions were predicted to form amphipathic helices (Figure 3A and Figure S3A). By comparison, none of the shorter MreBs were predicted to have any amphipathic helical regions (Figure S3A). When the N-terminal helix of EcMreB is plotted on a wheel, the hydrophobic residues cluster on one side of the helix, forming a predicted membrane-binding surface (Figure 3A). The two residues found to form the membrane insertion loop in TmMreB, L93 and F94, are not strictly conserved in all MreBs, although many have two hydrophobic residues nearby. In the case of EcMreB, residues F103 and M104 are positioned close to the equivalent TmMreB loop region, and we speculated that these may be required to facilitate membrane binding, although the amphipathic helix would probably be expected to contribute the majority of the membrane-binding energy.

### The Amphipathic Helix of EcMreB Is Sufficient to Localize GFP to the Membrane

In order to test whether the N terminus of EcMreB forms a true amphipathic helix, we constructed a GFP fusion protein that carried either one or two copies of the nine-residue N-terminal EcMreB peptide at its N terminus. As a positive control we constructed a GFP fusion protein that carried the known C-terminal amphipathic helix from *E. coli* MinD protein (Szeto et al., 2002) at the C terminus of GFP. As expected, GFP alone formed a diffuse localization pattern throughout the cell while the GFP-MinD helix fusion protein was localized to the membrane (Figure 3B). We found that GFP with one copy of the EcMreB amphipathic helix was only slightly localized at the membrane, but that when two copies were present in tandem the GFP was completely localized at the membrane (Figure 3B). This requirement for a double copy of the amphipathic helix has been observed with other amphipathic helices (Fischer et al., 2009) and reflects a weak binding energy that will be compensated in the cell by the many copies of amphipathic helices present along an MreB filament.

### EcMreB Binds Directly to Membranes, and This Is Mediated by an N-Terminal Amphipathic Helix

Despite extensive efforts, it has not been possible to purify the EcMreB protein in a state suitable for biochemical studies, and therefore we could not test for membrane binding using the same in vitro assays that we used to study TmMreB. We reasoned that this difficulty may be due to the hydrophobicity of the amphipathic helix, and indeed we were able to purify a stable mutant of EcMreB that lacked the N-terminal helix. This does not bind strongly to membranes, as shown by a vesicle-pelleting assay (Figure S3B), suggesting that the N terminus contributes the majority of the membrane binding energy. Indeed, the N-terminal helix (fused to GFP) does interact with vesicles, which becomes even more obvious when present in duplicate (Figure S3B). Due to the difficulties in purifying the full-length protein in a nonaggregated state, we turned to cellular experiments to test whether EcMreB also binds directly to membranes.

First, we performed the cellular overexpression experiments with EcMreB and observed cells containing high levels of EcMreB using electron cryotomography. Cells with high levels of wild-type EcMreB exhibited membrane invaginations and internal membrane-bound structures similar to those observed in cells containing high levels of TmMreB (Figure 3C). By comparison, cells containing high levels of EcMreB that lacked the N-terminal amphipathic helix showed no membrane invaginations, and large bundles of filaments were observed running along the length of the cell, not attached to membranes (Figure 3C). We found that cells containing the EcMreB mutant in the residues corresponding to the TmMreB membrane insertion loop (TmMreB, L93A/F94A; EcMreB, F103A/M104A, Figure S2) exhibited a somewhat unusual morphology in which filaments could be observed, similar to those from the N-terminal deletion, but in which membrane binding still occurred, similar to the wild-type. We suggest that this is due to the fact that the amphipathic helix continues to recruit EcMreB filaments to the membrane, as demonstrated by the GFP experiments (Figure 3B, Figure S3B), but that this structure is somewhat destabilized by the loss of hydrophobic residues in the loop that is forced into a position close against the membrane by the filament.

In order to further test membrane binding by EcMreB, we constructed an EcMreB-mCherry^SW^ fusion protein using an internal loop in EcMreB that was previously shown to be functional (Bendezú et al., 2009). We then studied the localization patterns of wild-type and membrane-binding mutants of this EcMreB-mCherry^SW^ fusion protein when it was expressed in wild-type cells at low levels using leaky expression from a *tac* promoter (Figure 3D). Cells are sensitive to small overexpression or depletion of MreB. Therefore despite the low expression levels we found that cells carrying wild-type EcMreB-mCherry^SW^ were generally slightly rounded and contained membrane invaginations as shown by the green FM1-43 membrane dye (Figure 3D). EcMreB-F103A/M104A-mCherry^SW^ mutants were also rounded with distorted membrane staining, supporting our analysis that the amphipathic helix is sufficient to confer most, if not all, membrane-binding activity. However, when the N-terminal amphipathic helix was removed, both with and without the additional membrane insertion loop mutation, the FM1-43-stained membrane invaginations completely disappeared and long bundles of EcMreB-mCherry^SW^ filaments could be observed running along the length of the cell (Figure 3D, compare with Figure 3C). There was no residual localization of EcMreB-mCherry^SW^ anywhere close to the edge of the cell. Taken together, these results show that wild-type EcMreB directly binds membranes and can form invaginations when present at high levels, and that this membrane binding is predominantly achieved by the short N-terminal amphipathic helix.

### Membrane Binding Is Essential for the Function of EcMreB

**Figure 4:**
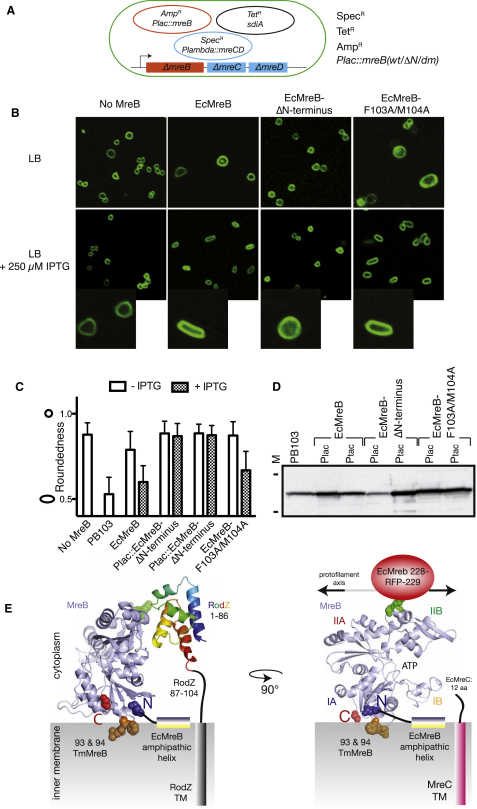
The Amphiphatic Helix of MreB Is Required for Cell Shape Maintenance in *E. coli* (A) Schematic diagram showing the genetic background. An MreBCD knockout strain (*mreBCD* < > *frt*, FB17) carries three plasmids: a plasmid constitutively expressing transcription factor SdiA (pFB112, tet^R^) that enhances the expression of FtsQAZ, a plasmid that carries *mreCD* downstream of a lambda promoter and temperature-sensitive repressor (pFB124, spec^R^), and a plasmid expressing the *mreB* variants under a *lac* promoter (pFB209, wild-type MreB; pFE379, EcMreB-ΔN terminus; pFE380, EcMreB-F103A/M104A, all amp^R^). (B) The N-terminal amphipathic helix of MreB is essential for shape maintenance in *E. coli*. Confocal microscopy images of strain FB17/pFB112/pFB124 (*ΔmreBCD/tet sdiA/ aadA clts plambda::mreCD*, first column) and of the same strain transformed with pFB209 (*plac::EcmreB*, second column), pFE379 (*plac::EcMreB-ΔN*, third column), and pFE380 (*plac::EcMreB-F103A/M104A*, fourth column). MreB versions were induced with 250 μM IPTG. Cells that express functional MreB (EcMreB and EcMreB-F103A/M104A) revert to rod shape, whereas expression of EcMreB-ΔN results in misshapen cells. Cells were grown for 6–7 hr in LB at 37°C supplemented with ampicillin and spectinomycin and stained with FM4-64 prior to visualization with a Zeiss confocal laser scanning microscope LSM 510. (C) Cell shape distribution. The graph presents width/length ratios as a measure of cell roundedness (performed computationally with ImageJ). Perfect round cells have a value of 1.0 and perfect rods a value around 0.6 for *E. coli*. Strains and growth conditions were the same as in (B), and strain PB103 was used as a wild-type control. The graph is based on three independent experiments, with total number of cells measured as follows: no MreB (FB17/pFB112/pFB124), n = 114; PB103, n = 87; EcMreB (FB17/pFB209/pFB124), n = 76 (−IPTG), n = 82 (+IPTG); Plac::EcMreB-ΔN (FB17/pFE379/pFB124), n = 131 (−IPTG), n = 150 (+IPTG); Ptac::EcMreB-ΔN (FB17/pFE377/pFB124), n = 106 (−IPTG), n = 52 (+IPTG); EcMreB-F103A/M104A (FB17/pFE380/pFB124), n = 98 (−IPTG), n = 143 (+IPTG). Error bars represent standard deviations. (D) Western blot showing the levels of MreB variants in extracts from the corresponding strains used in Figure 4C. Equal amounts of cells were loaded and MreB was detected using affinity-purified α-MreB antibodies. The positions of the 35.9 and 52.7 kDa standards are indicated. (E) Schematic diagram showing the position of MreB on the membrane and its interactions with RodZ (known from a cocrystal structure, van den Ent et al., 2010) and the C-terminal peptide of EcMreC. The membrane insertion loop and EcMreB amphipathic helix are shown. The position of mCherry^SW^ used to construct a functional fusion protein in the internal loop is shown, and the close positions of the N and C termini to the membrane reveal why previous N- or C-terminal fusion proteins were likely nonfunctional.

To determine if the observed membrane binding of MreB is required for cell shape maintenance and viability, we studied the phenotypes of membrane-binding mutants of EcMreB in vivo. EcMreB (wild-type), EcMreB-F103A/M104A (double mutant), or EcMreB-ΔN (lacking the N-terminal amphiphatic helix) was expressed from a moderate copy number plasmid (controlled by a *lac* promoter) in Δ*mreBCD* strain FB17. Since all three Mre proteins are essential for rod-shape maintenance and normal cell viability, the strain harbored two additional plasmids, pFB124 and pFB112 (Figure 4A). The former expresses MreC and MreD in a temperature-sensitive manner, and the latter constitutively expresses the transcription factor SdiA, ensuring elevated levels of FtsQAZ that are required and sufficient for spherical cells to survive and propagate (Bendezú and de Boer, 2008). The phenotypes of cells producing the different MreB versions were then investigated and compared to that of cells not producing any MreB (EcMreB^−^). A first indication of the importance of the N-terminal amphipathic helix for cells to survive came from the observation that, like EcMreB^−^ cells, EcMreB-ΔN cells required the *sdiA* plasmid to survive (Figure S4A). In contrast, cells producing wild-type protein or EcMreB-F103A/M104A rapidly lost the SdiA expression plasmid when grown in the absence of selective antibiotics (Figure S4A), indicating that the double substitution did not abrogate MreB function as severely as removal of the N-terminal helix.

In the absence of MreB, cells are spherical (top row, Figure 4B, quantified in Figure 4C), and they revert to rod shape upon production of wild-type EcMreB (Figure 4B, second column, Figure 4C), (Bendezú and de Boer, 2008). In contrast, EcMreB-ΔN protein fails to correct cell shape, and cells remain spherical in the presence of inducer. Quantification of cell roundness (length of minor axis divided by length of major axis) shows that EcMreB-F103A/M104A behaves similarly, but not identically, to the wild-type protein (Figure 4C). Upon IPTG induction, cells revert back to rod shape but remain slightly fatter than cells producing the wild-type protein. This might indicate that the double mutation has a minor effect on cell shape, but it is clearly not as dramatic as removal of the N-terminal amphipathic helix. MreB expression levels were assayed by immunoblot analysis (Figure 4D) and found to be lower for EcMreB-ΔN than the other two proteins when induced under control of the *lac* promoter. To ensure that this difference did not cause the observed difference in cell shape, the experiment was repeated using the stronger *tac* promoter to drive MreB production. This confirmed that EcMreB-ΔN was nonfunctional, as it failed to correct cell shape even when produced at several-fold the level of EcMreB in wild-type cells (Figures 4C and 4D). We conclude that the N-terminal amphipathic helix of EcMreB is essential for its function in cell shape maintenance and viability.

## Discussion

Here we show that bacterial actin MreB from two organisms, *E. coli* and *T. maritima*, interacts directly with the cell membrane, and we predict that this will be applicable to all other MreBs (Figure 2D and Figure S3A). We propose that all MreBs bind to membranes via an insertion loop and/or an amphipathic helix and that the orientation of the MreB filaments relative to the membrane is conserved (Figure 2D).

This unexpected discovery explains a number of previous observations, such as the finding that N- and C-terminal GFP fusions of MreB are not fully functional. This can now be explained as both ends are positioned close to the membrane where these fusion proteins will interfere with essential membrane binding (Figure 4E). Also, MreB has been shown to associate with membrane proteins MreC, MreD, and RodZ (Kruse et al., 2005; van den Ent et al., 2010; White et al., 2010). The exact interaction site with RodZ is known from a cocrystal structure (van den Ent et al., 2010), and satisfyingly, the RodZ binding site and linker peptide are fully compatible with the membrane binding model of the filament proposed in Figure 2D (Figure 4E). The integral membrane protein MreD has short stretches of six to ten amino acids that are exposed to the cytoplasm and the bitopic membrane protein MreC also only contains a rather short cytosolic peptide (nine residues for *E. coli* MreC). Only when the MreB filament is positioned flush against the cell membrane can all these interaction partners be brought within suitable reach of one another (Figure 4E).

The finding that MreB interacts directly with the cell membrane raises some interesting questions. Membrane binding does not strictly require nucleotide binding, as demonstrated here, although it is possible that the nucleotide state is involved in modulating membrane-binding affinities. Filamentation occurs upon nucleotide binding, and as MreB is anchored in the membrane it will immediately compartmentalize the inner membrane if the filaments were long enough to impose a barrier to lateral diffusion of both integral and peripheral membrane proteins. Recent evidence suggests that MreB filaments are most likely composed of rather short filaments (Swulius et al., 2011), although even short filaments could still provide organization to the membrane through their barrier function.

EcMreB now joins a family of cytoskeletal prokaryotic proteins (including MinD and FtsA; Szeto et al., 2002; Pichoff and Lutkenhaus, 2005) that carry an amphipathic helix, and to our knowledge, this feature has so far not been described for any eukaryotic filament system. Polymerization kinetics might be partially restricted to two rather than three dimensions, and consequently the critical concentration for filament formation is likely lower than if it were not attached to the membrane.

Our data lead us to two competing structural models for TmMreB filaments. The electron microscopy images reveal that TmMreB assembles into double filaments, as has been shown previously (Esue et al., 2005; Popp et al., 2010; van den Ent et al., 2001). Although it is not possible to determine the exact orientation of MreB filaments from these data, the closed symmetry of the doublets, together with the position of the membrane insertion loops, results in structural constraints that can best be satisfied when TmMreB assembles into pairs of protofilaments that are aligned either in an antiparallel orientation (Figure S4B, left) or in a parallel orientation but where the two membrane binding surfaces are on opposite sides (Figure S4B, right). The alternative orientation of parallel protofilaments arranged front to back (Figure S4B, center) is not in agreement with the EM reconstruction and is unlikely as lateral growth would be unlimited. The antiparallel model best satisfies the membrane-binding demands of the insertion loops but would be surprising, as it would differ from all known actin-like filaments. In both models the inner surface of the TmMreB doublet matches the inner surface of both actin (right-handed double helical) and ParM filaments (left-handed double helical), preserving an important structural feature between the filaments of all actin-like proteins.

## Experimental Procedures

### Strains and Plasmids

Strains, plasmids, and growth conditions are detailed in the Supplemental Experimental Procedures and in Table S1.

### Protein Purification

TmMreB was purified both as a His-tagged protein and as an untagged protein using a cleavable intein fusion. Detailed descriptions are given in the Supplemental Experimental Procedures.

### Preparation of 2D Lipid Monolayers and Negative Stain Electron Microscopy

2D lipid monolayers were prepared following the protocol of Kelly et al. (2008). Briefly, a lipid monolayer composed of *E. coli* polar lipid extract (Avanti Polar Lipids) was formed on the surface of a carbon-coated electron microscopy nickel grid. TmMreB was incubated in a droplet exposed to the monolayer at 0.2 mg/ml in polymerization buffer (100 mM Tris [pH 7.4], 100 mM NaCl, 3 mM MgCl_2_, 1.5 mM AMP-PNP). The sample was stained using 2% uranyl acetate and imaged using a 120 kV Tecnai 12 electron microscope (FEI Company, Eindhoven, NL).

### Vesicle Preparation

Vesicles used in pelleting assays and electron microscopy experiments were prepared using *E. coli* total lipid extract (Avanti Polar Lipids). Vesicles were prepared in TEN buffer (50 mM Tris, 1 mM EDTA, 200 mM NaCl [pH 7.4]) using sonication and extrusion, with a pore size of 1 μm.

### Pelleting Assays

Prespun MreB was mixed with vesicles in TEN buffer in a volume of 100 μl, with a final MreB concentration of 1 mg/ml and lipid concentration of 0.5 mg/ml. Samples were incubated at room temperature for 15 min, then spun at 40,000 rpm in a Beckman TLA 100 rotor at 25°C.

### Preparation of MreB-Bound Vesicles and Electron Cryomicroscopy

Vesicles were mixed with 1 mg/ml MreB in polymerization buffer and immediately frozen on grids for electron cryomicroscopy.

### Protein Overexpression and Cellular Electron Cryotomography

Protein overexpression experiments were performed using the T7 expression system in BL21 *E. coli* cells. Induced cells were frozen directly from the growth media on Quantifoil (Quantifoil Micro Tools) grids. Electron tomography was performed using an FEI Tecnai G2 Polara (300 kV, liquid nitrogen cooling, FEI).

### Fluorescence Light Microscopy

*E. coli* cell strain TG1tr (EcMreB-mcherry^SW^ constructs) or C41 (GFP constructs) was used. Cells were taken from the growth media and placed on an LB-agar pad containing FM4-64 or FM1-43 dye, and imaged at room temperature within 2 hr. Microscopy was performed using a Zeiss LSM510 confocal laser-scanning microscope, using a 63× oil immersion objective lens.

### Genetic Complementation Experiments

Mutant versions of MreB were expressed from a *lac* promoter on a low copy number plasmid in an MreBCD knockout strain FB17 (Bendezú and de Boer, 2008), and their phenotype was examined. The MreBCD knockout strain additionally carried pFB112 (tet^R^) that constitutively expresses SdiA and pFB124 (spec^R^) that expresses MreCD at 37°C (Bendezú and de Boer, 2008). Cells were stained with FM4-64 and examined with a Zeiss laser-scanning confocal microscope (LSM510) and processed with ImageJ.

## Supplemental Information

Document S1. Four Figures, Supplemental Experimental Procedures, Two Tables, and Supplemental References

Movie S1. 3D Electron Cryotomography Reconstruction of a Rapidly Frozen *E. coli* Cell, Overexpressing High Levels of TmMreB TmMreB induces membrane invaginations and curvature, and patches of apparent attachment between membrane surfaces can be observed at the edges of the cell. Related to Figure 1.

Movie S2. 3D Electron Cryotomography Reconstruction of Lipid Vesicles in the Presence of TmMreB and AMP-PNP Extensive patches of attachment between adjacent membranes can be observed. Double filaments of TmMreB filaments can be resolved, and the double bilayer in an internal, protected liposome is visible. Related to Figure 2.
