# Supplementary material for: Direct Membrane Binding by Bacterial Actin MreB
Source: Mol Cell. 2011 Aug 5;43(3-6):478–87. doi: 10.1016/j.molcel.2011.07.008 (PMC3163269; doi:10.1016/j.molcel.2011.07.008)
Supplement: Document S1. Four Figures, Supplemental Experimental Procedures, Two Tables, and Supplemental References [file mmc1.pdf]

Molecular Cell, Volume *43*

Supplemental Information

Direct Membrane Binding by Bacterial Actin MreB

Jeanne Salje, Fusinita van den Ent, Piet de Boer, and Jan Löwe

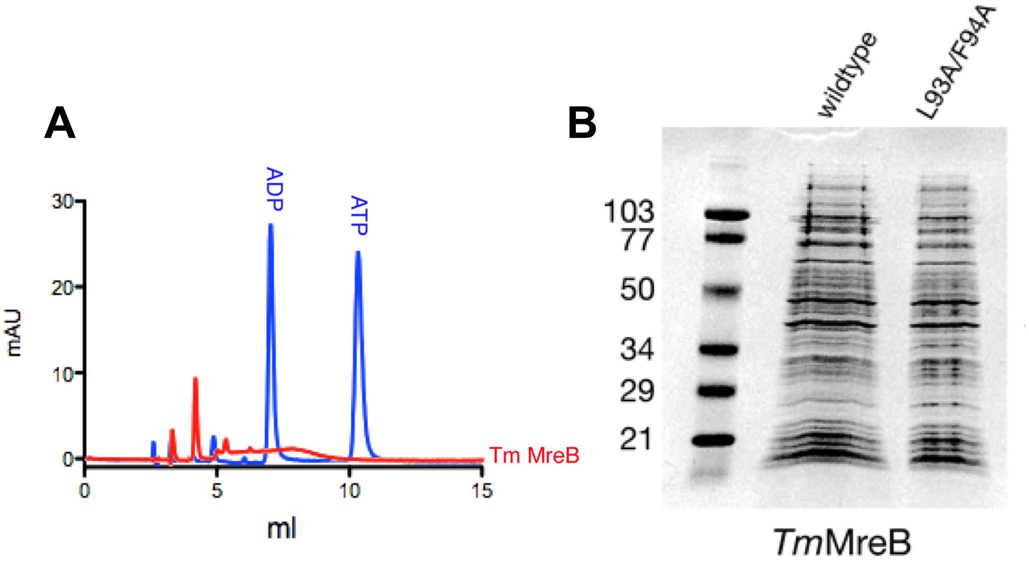

Figure S1, related to main figure 1: A. Purified *T. maritima* MreB does not contain nucleotide: 5 nmol of purified protein was denatured with 10% aceton, spun and the supernatant was injected onto a C18 column directly after nucleotide standards were run (5 nmol of ADP and ATP). The column was run in TBAB buffer, pH 6.5 (33 mM K_2HPO_4, 67 mM KH_2PO_4, 10 mM tetrabutylammoniumbromide, 7.5% acetonitril). **B.** Expression gels from cells used in electron cryotomography. Whole cell SDS-PAGE gel shows overexpression of wildtype (TmMreB, pFE309) and TmMreB (TmMreB_L93A/F94A, pJS101).

```
EcMreB    1 mlkkfrgmfsndlsidlgtantliyvkgqgivlnepsvvairqdragspk  50
                   |...|:.|||||||||::::|:|||:|||||:||    ..:..
TmMreB1   1 -------mlrkdigidlgtantlvflrgkgivvnepsviai----dsttg  39

EcMreB   51 svaavghdakqmlgrtpgniaairpmkdgviadffvtekmlqhfikqvhs 100
            .:..||.:||.|:|:||..|.|||||:||||||:.|...||::||.:...
TmMreB1  40 eilkvgleaknmigktpatikairpmrdgviadytvalvmlryfinkakg  89

EcMreB  101 --nsfmrpsprvlvcvpvgatqverrairesaqgagarevflieepmaaa 148
              |.|   .|||::.||:|.|.||||||.::...|||.:|||||||||||
TmMreB1  90 gmnlf---kprvvigvpigitdverraildagleagaskvflieepmaaa 136

EcMreB  149 igaglpvseatgsmvvdigggttevavislngvvysssvriggdrfdeai 198
            ||:.|.|.|.:|:|||||||||||||||||..:|...|:||.||..||||
TmMreB1 137 igsnlnveepsgnmvvdigggttevavislgsivtwesiriagdemdeai 186

EcMreB  199 inyvrrnygsligeataerikheigsaypgdevreie--vrgrnlaegvp 246
            :.|||..|...|||.||||:|.|||:.:|..|..|:|  |.|.:|:.|:|
TmMreB1 187 vqyvretyrvaigertaervkieignvfpskendelettvsgidlstglp 236

EcMreB  247 rgftlnsneilealqepltgivsavmvaleqcppelasdisergmvltgg 296
            |..||...|:.|||:..:..||.:|...||:.||||.|||.|||:.||||
TmMreB1 237 rkltlkggevrealrsvvvaivesvrttlektppelvsdiiergifltgg 286

EcMreB  297 gallrnldrllmeetgipvvvaedpltcvargggkalemid----mhggd 342
            |:|||.||.||.:||||.|:.:|:|||.||:|.|..|:.::    :.|..
TmMreB1 287 gsllrgldtllqketgisvirseepltavakgagmvldkvnilkklqgag 336

EcMreB  343 lfsee    347

TmMreB1 336 -----    336
```

Figure S2, related to main figure 2: Sequence alignment between EcMreB and TmMreB. Residues marked in yellow indicate membrane-binding sequences. Residues in red mark the insertion site of RFP (codon-optimised mCherry), a functional sandwich fusion in EcMreB (Bendezú *et al.,* 2009, *EMBO Journal* **28**, 193-204).

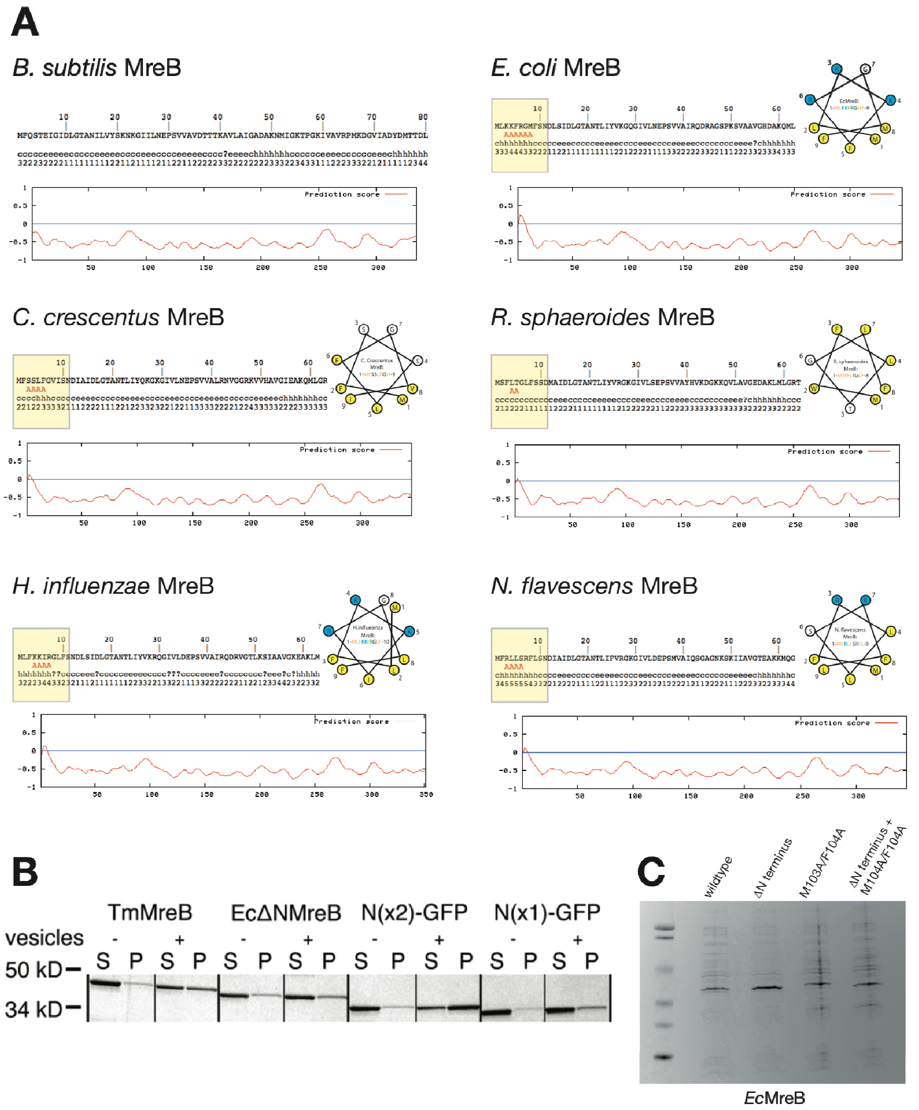

Figure S3, related to main figure 3: A. Amphipathic helix prediction of MreBs. Results from the amphipathic helix prediction software AMPHIPASEEK (http://npsa-pbil.ibcp.fr/cgi-bin/npsa_automat.pl?page=/NPSA/npsa_amphipaseek.html) for MreB from Gram positive (*B. subtilis)* and Gram negative (*E. coli, C. crescentus, R. sphaeroides, H. influenzae* and *N. flavescens*) bacteria. Predicted in-plane membrane anchors are indicated by red 'A's, and marked by a yellow boxed area. The helical wheel for the first 8 or 9 residues shows hydrophobic residues in yellow, positively charged residues in blue and neutral residues on a white background. **B.** Vesicle pelleting assay showing that the N-terminal helix of EcMreB binds membranes. Proteins used: TmMreB (pFE52), EcMreB lacking the N-terminal helix (EcΔNMreB, pFE355), N-terminal helix of EcMreB fused to GFP in one copy (pFE356) or in two copies (pJS111). Protein concentration was 27 µM, vesicle concentration was at 0.5 mg/ml, in TEN200, pH 8.0, supplemented with 3 mM TCEP. **C.** Expression gels from cells used in electron cryotomography. Whole cell SDS-PAGE gel shows overexpression of wildtype (EcMreB, pFE57) and mutant EcMreB (EcMreB_ΔN-terminus, pJS108, EcMreB_F103A/M104A, pJS107, EcMreB_ΔN-terminus_ F103A/M104A, pJS109).

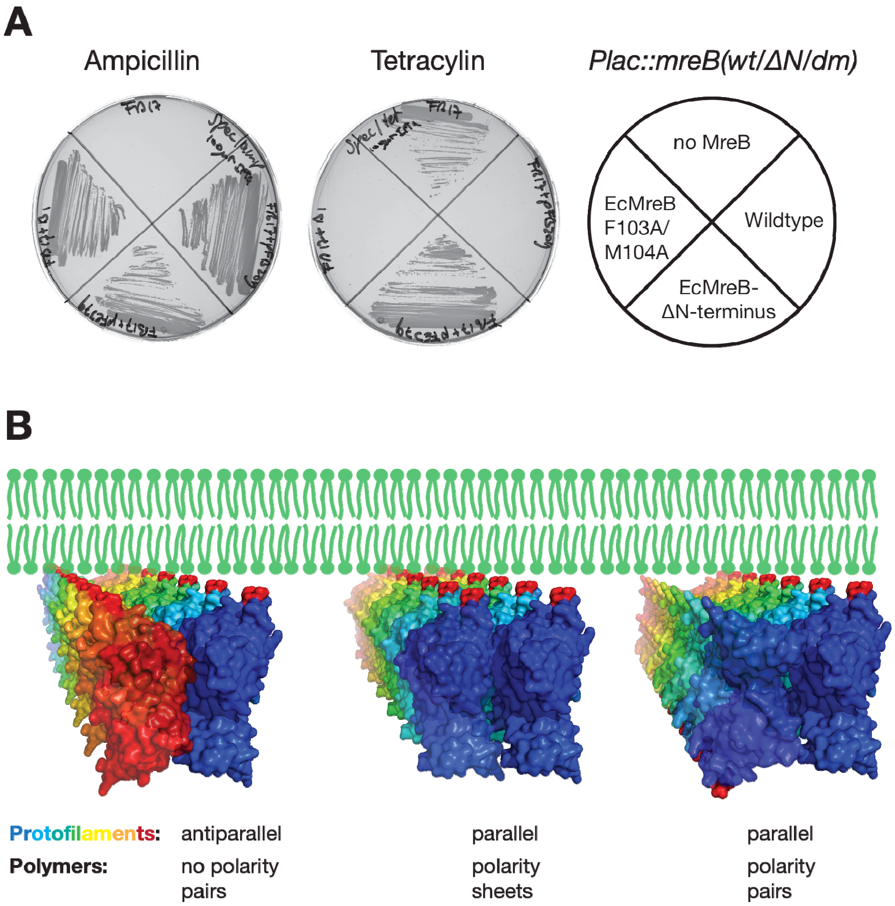

Figure S4, related to main figure 4: A. The N-terminal amphipathic helix is required for cell viability. The MreB knockout strain needs either extra FtsQAZ or functional MreB to survive (Bendezú and de Boer, 2008). In the presence of functional MreB and the absence of tetracycline, cells will become rod shaped and tetracycline sensitive as the MreB plasmid will compete out pFB112 (SdiA, which enhances expression of *ftsZ*). Cells grown in the absence of tetracycline expressing wildtype MreB (pFB209) or EcMreB-F103A/M104A (pFE380) rapidly become tetracycline sensitive. In the absence of an *mreB*-containing plasmid or upon expression of EcMreB-ΔN (pFE379), cells remain tetracycline resistant, as they continue to require the SdiA expressing plasmid. Cells were grown in LB at 37°C in the presence of ampicillin and spectinomycin. **B.** Models for different possible arrangements of MreB protofilaments. The important variables are the relative orientation of individual subunits (parallel or antiparallel) and the relative direction of adjacent protofilaments (parallel or antiparallel). Each protofilament is coloured in rainbow colours and a small protrusion in red indicates the membrane insertion loop/amphipathic helix. Our EM reconstruction suggest pairs of protofilaments (as drawn on the left and right side) rather then sheets of individual filaments (which would be the result of the central model). Members of the actin family of proteins F-actin and ParM adopt a parallel arrangement of protofilaments as suggested on the right.

Supplemental Movies

Movie S1, related to main figure 1: 3D electron cryotomography reconstruction of a rapidly frozen *E. coli* cell, overexpressing high levels of TmMreB. TmMreB induces membrane invaginations and curvature, and patches of apparent attachment between membrane surfaces can be observed at the edges of the cell.

Movie S2, related to main figure 2:. 3D electron cryotomography reconstruction of lipid vesicles in the presence of TmMreB and AMP-PNP. Extensive patches of attachment between adjacent membranes can be observed. Double filaments of TmMreB filaments can be resolved, and the double bilayer in an internal, protected liposome is visible.

Supplemental Experimental Procedures

Strains and plasmids

All plasmids are listed in Table SI. The *E. coli* strain C41(DE3) (Miroux and Walker, 1996) was used for all protein expression and cellular electron cryotomography experiments. Light microscopy was carried out on *E. coli* TG1tr cells, except for the complementation experiments for which a *E. coli* PB103 cells and a derivative (FB17) were used (Bendezú and de Boer, 2008). Proteins were non-tagged for cellular electron cryotomography and light microscopy and were his-tagged when purified, unless stated otherwise. Cells were grown in Luria Broth media at 37 °C unless otherwise described. Cells were grown in the presence of antibiotic (100 μg/ml ampicillin for high copy number plasmids, 50 μg/ml ampicillin for low copy number plasmids, 12.5 μg/ml tetracycline, 50 μg/ml spectinomycin) as appropriate.

Protein purification

Protein expression and purification was performed as described previously for the His-tagged proteins (van den Ent et al., 2001). Non-tagged MreB from *T. maritima* (pFE349) was purified as an intein fusion linked to chitin binding domain (NEB). The protein was expressed at 37 °C in C41 cells for 5 hours. Cells were lysed in buffer A (20 mM Tris pH 8.5, 500 mM NaCl, 0.1 mM EDTA), supplemented with DNase (Sigma) and protease inhibitor tablets (Roche) by passing it through a cell disruptor (Constant Systems) at 20 kpsi. The cleared lysate was passed over a chitin column (NEB) that was subsequently washed with 4 column volumes of buffer A and 3 column volumes buffer B (50 mM Tris pH 8.5, 200 mM NaCl, 0.1 mm EDTA, 50 mM DTT). The fusion protein was cleaved overnight at 4 °C. Non-tagged MreB was eluted from the column in buffer B, concentrated and further purified over Sephacryl S200 in TEN200 buffer (20 mM Tris pH 7.5, 1 mM EDTA, 1 mM sodium azide and 200 mM NaCl). Peak fractions were concentrated and stored frozen at 7.4 mg/ml. EcMreB lacking the N-terminal helix fused to intein-chitin binding domain (pFE355) was purified as described for pFE349, with buffer A being (20 mM Tris pH 8.5, 500 mM NaCl, 1 mM EDTA, 1 mM TCEP) and buffer B (20 mM Tris pH 8.0, 200 mM NaCl, 0.1 mM EDTA, 50 mM DTT). The protein was eluted from Sephacryl S200 column in TEN200, pH 8.0 (20 mM Tris pH 8.0, 1 mM EDTA, 1 mM sodium azide), supplemented with 3 mM TCEP. Peak fractions were concentrated and stored at 8 mg/ml.

Plasmid descriptions:

Primer sequences are listed in Table S2.

pFE349 (TmMreB-intein-chitin_binding_domain) was constructed as follows: the *mreB* gene (SWALL: Q9WZ57) was amplified by PCR using the primers inttmbF and inttmbR, from genomic DNA (DSMZ number 3109). The construct was cloned using In-Fusion (Clontech) according to the manufacturer protocol with gelpurified insert and vector (pTXB1 (NEB)), resulting in no extra residues between *mreB* and *intein*.
pFE309 (TmMreB): The gene of *tmmreb* was amplified using primers Tmb1F and TmbstopR. The PCR product was cleaved with NdeI/BamHI and ligated to NdeI/BamHI digested pOPT (obtained from O. Perisic, MRC-LMB, Cambridge, UK).
pJS100 (TmMreB_L93A) pFE309 was used as a template for QuickChange mutagenesis (Stratagene) using primers TmL93AF and TmL93AR.
pJS101 (TmMreB_L93A/F94A) pJS100 was used as a template for QuickChange mutagenesis (Stratagene) using primers TmL93AF94AF and TmL93AF94AR.
pJS103 (TmMreB_D153A) pFE309 was used as a template for QuickChange mutagenesis (Stratagene) using primers TmD153AF and TmD153AR.
pJS104 (TmMreB_L93A_6His) pFE52 was used as a template for QuickChange mutagenesis (Stratagene) using primers TmL93AF and TmL93AR.
pJS105 (TmMreB_L93A/F94A_6His) pJS104 was used as a template for QuickChange mutagenesis (Stratagene) using primers TmL93AF94AF and TmL93AF94AR.
pFE57 (EcMreB) The gene of *ecmreb* was amplified using primers EmbF and EmbR. The PCR product was cleaved with NdeI/BamHI and ligated to NdeI/BamHI digested pHis17 (B. Miroux personal communication).
pJS107 (EcMreB_F103A/M104A) pFE57 was used as a template for QuickChange mutagenesis (Stratagene) using primers EcF103AM104AF and EcF103AM104AR.
pJS108 (EcMreB_ΔN) pFE57 was used as a template for QuickChange mutagenesis (Stratagene) using primers EmbdeltaNF and EmbdeltaNR.
pJS109 (EcMreB_ΔN_F103A/M104A) pJS107 was used as a template for QuickChange mutagenesis (Stratagene) using primers EmbdeltaNf and EmbdeltaNr.
pFE355 (EcMreB_ΔN-intein-chitin_binding_domain). A PCR product was obtained with primers EmbdeltaNinteinF and EmbinteinR and cloned into pTXB1 (Invitrogen) using In-Fusion (Clontech).
pFE356 (Em_GFP-1xN-terminus from EcMreB). QuickChange mutagenesis (Stratagene) on pRSET/EmGFP (Invitrogen) was done using NembgfpF and NembgfpR primers.
pJS110 (Em_GFP-C-terminus from EcMinD) The C-terminal peptide of MinD was fused to the C-terminus of GFP by QuickChange mutagenesis (Stratagene) using pRSET/EmGFP (Invitrogen) as template and primers GFPMinDf and GFPMinDr.
pJS111 (Em_GFP-2xN-terminus from EcMreB) The N-terminal peptide of EcMreB was fused to the N-terminus of GFP by two rounds of QuickChange mutagenesis (Stratagene) using pRSET/EmGFP (Invitrogen) as template and primers GFPEcMreB1F and GFPEcMreB1R and then GFPEcMreB2F and GFPEcMreB2R.
pFE363 (EcMreB-mcherry^{SW}). To clone EcMreB-mcherry^{SW} into a low copy number plasmid under the control of tac promoter, the insert was amplified using EmbF and EmbR primer from pFE359 (EcMreB-mcherry^{SW} in pHis17) then digested with NdeI and BamHI and ligated into NdeI/BamHI digested pMal-c2x (NEB). pFE359 was

obtained in several steps. The gene for *mcherry* was amplified from pRod17, a codon optimised version for expression in *E. coli* (D. Sheratt personal communication). Primers embintmc4 and embintmcherryR2 partially overlap with 27 bp of mreB sequence adjacent to the integration site. The PCR product was used as a primer pair in RF cloning (van den Ent and Löwe, 2006) on pFE57, obtaining a sandwich fusion where SGSS-mCherry-SGAPG is inserted after G228 of EcMreB, previously shown to be functional {Bendezú et al., 2009, EMBO J, 28, 193-204}.

pFE364 (EcMreB-ΔN F103A/M104A-mcherry^{SW}). The insert was amplified using EmbdeltaN and EmbR on pFE362 then digested with NdeI and BamHI and ligated into NdeI/BamHI digested pMal-c2x (NEB). pFE362 was obtained by QuickChange mutagenesis (Stratagene) using two primer pairs: EmbdeltaNF and EmbdeltaNR and EmbdmF and EmbdmR on EcMreB-mcherry^{SW} in pHis17.

pFE365 (EcMreB-F103A/M104A-mcherry^{SW}). The insert was amplified using EmbF and EmbR primer on pFE361. pFE361 was obtained by QuickChange using the primer pair EmbdmF and EmbdmR on EcMreB-mcherry^{SW} in pHis17.

pFE366 (EcMreB-ΔN-mcherry^{SW}). The insert was amplified using EmbdeltaN and EmbR on pFE360 then digested with NdeI and BamHI and ligated into NdeI/BamHI digested pMal-c2x (NEB). pFE360 was obtained by QuickChange mutagenesis (Stratagene) using the primer pair EmbdeltaNF and EmbdeltaNR on EcMreB-mcherry^{SW} in pHis17.

pFB209 (plac::EcMreB) has been described before (Bendezú et al., 2009).

pFE379 (plac:: EcMreB-ΔN): The primerpair dN209F and dN209R was used in Quickchange mutagenesis on pFB209 (plac::EcMreB).

pFE380 (plac:: EcMreBF103A/M104A) was constructed by overlapping PCR using pFB209ecoF and embdmR primer on pFB209 to obtain a 443 bp PCR product that then was used in a second PCR together with pFB209HindR to generate an 1170 bp insert. Insert and pFB209 were cleaved with EcoRI and HindIII, purified and ligated to obtain pFE380.

All constructs were verified by sequencing.

Table S1

Plasmid	Description	tag	Resistance	Promoter	Vector	Reference
pFE349	*T. maritima* MreB	Intein-CBD	ampicillin	T7	pTXB1	this work
pFE309	*T. maritima* MreB	No	ampicillin	T7	pOPT	this work
pJS100	*T. maritima* MreB_L93A	No	ampicillin	T7	pHis17	this work
pJS101	*T. maritima* MreB_L93A/F94A	No	ampicillin	T7	pHis17	this work
pJS103	*T. maritima* MreB_D153A	No	ampicillin	T7	pHis17	this work
pFE52	*T. maritima* MreB	Yes	ampicillin	T7	pHis17	(van den Ent et al., 2001)
pJS104	*T. maritima* MreB_L93A	Yes	ampicillin	T7	pHis17	this work
pJS105	*T. maritima* MreB_L93A/F94A	Yes	ampicillin	T7	pHis17	this work
pFE57	*E. coli* MreB	No	ampicillin	T7	pHis17	this work
pJS107	*E. coli* MreB_F103A/M104A	No	ampicillin	T7	pHis17	this work
pJS108	*E. coli* MreB_ΔN-terminus	No	ampicillin	T7	pHis17	this work
pJS109	*E. coli* MreB_ F103A/M104A + ΔN-terminus	No	ampicillin	T7	pHis17	this work
pFE355	*E. coli* MreB_ΔN-terminus	Intein-CBD	ampicillin	T7	pTXB1	this work
pRSET/ EmGFP	Em_GFP	Yes	ampicillin	T7	pRSET	Invitrogen
pFE356	Em_GFP + 1x N-terminus from *E. coli* MreB	Yes	ampicillin	T7	pRSET	this work
pJS110	Em_GFP+C-terminus from MinD	Yes	ampicillin	T7	pRSET	this work
pJS111	Em_GFP+ 2x N-terminus from *E. coli* MreB	Yes	ampicillin	T7	pSET	this work
pFE363	*E. coli* MreB-mcherry^{SW}	No	ampicillin	Ptac	pMal-c2x	this work
pFE364	*E. coli* MreB-ΔN F103A/M104A-mcherry^{SW}	No	ampicillin	Ptac	pMal-c2x	this work
pFE365	*E. coli* MreB-F103A/M104A-mcherry^{SW}	No	ampicillin	Ptac	pMal-c2x	this work
pFE366	*E. coli* MreB-ΔN mcherry^{SW}	No	ampicillin	Ptac	pMal-c2x	this work
pFB209	*E. coli* MreB	No	ampicillin	Plac	pMLB1113	(Bendezú and de Boer, 2008).
pFB112	sdiA	No	tetracyclin	consititutive	pBR322	(Bendezú and de Boer, 2008).
pFB124	*E. coli* MreC, MreD-LE	No	spectinomycin	λP_R	pZC100	(Bendezú and de Boer, 2008).
pQW120	*E. coli* MreB	No	ampicillin	Ptac	pMal-c2x	C. Wang pers. communication
pFE377	*E. coli* MreB_ΔN-terminus	No	ampicillin	Ptac	pMal-c2x	this work
pFE378	*E. coli* MreB_ΔN-F103A/M104A	No	ampicillin	Ptac	pMal-c2x	this work
pFE379	*E. coli* MreB_ΔN-terminus	No	ampicillin	Plac	pMLB1113	this work
pFE380	*E. coli* MreB-F103A/M104A	No	ampicillin	Plac	pMLB1113	this work

Note that with the exception of pFB124 (a pSC101 derivative) all plasmids are derived of ColE1.

Table S2

Primer	Sequence
inttmbF	5′-CTTTAAGAAGGAGATATACATATGTTGAGAAAAGACATAGGAATAG
inttmbR	5′-GCATCTCCCGTGATGCACCCGGCACCCTGAAGCTTC
Tmb1F	5′-AGTCTACCATATGTTGAGAAAAGACATAGGAATAGATCTC
TmbstopR	5′-GGCATAAGGATCCTCACCCGGCACCCTGAAGCTTCTTCAGAATG
EmbF	5′-AGTCTACCATATGTTGAAAAAATTTCGTGGCATGTTTTCCAATG
EmbR	5′-TGACTACGGATCCTTACTCTTCGCTGAACAGGTCGCCGCCGTGCATGTCG
EmbdeltaNinteinF	5′-CTTTAAGAAGGAGATATACATATGTTTTCCAATGACTTGTCCATTG
EmbinteinR	5′-GCATCTCCCGTGATGCACTCTTCGCTGAACAGGTCGC
NembgfpF	5′-GAAGGAGATATACATATGTTGAAAAAATTTCGTGGCATGTTTCGGGGTTCTCATCATC
NembgfpR	5′-GATGATGAGAACCCCGAAACATGCCACGAAATTTTTTCAACATATGTATATCTCCTTC
Embintmc4	5′-CACGAAATCGGTTCGGCTTATCCGGGCTCAGGGTCCTCGGTGAGCAAGGGCGAGGAGGATAA
EmbintmcherryR2	5′-CACGAACTTCGATTTCACGGACTTCATCTCCGGGCGCTCCGGACTTGTACAGCTCGTCCATGCCAC
EmbdeltaNF	5′-CTTTAAGAAGGAGATATACATATGTTTTCCAATGACTTGTCCATTG
EmbdeltaNR	5′-CAATGGACAAGTCATTGGAAAACATATGTATATCTCCTTCTTAAAG
EmbdmF	5′-CAAGTGCACAGCAACAGCGCTGCGCGTCCAAGCCCGCGCG
EmbdmR	5′-CGCGCGGGCTTGGACGCGCAGCGCTGTTGCTGTGCACTTG
promdNF	5′-CAGGATTATCCCTTAGTATGTTTTCCAATGACTTGTCCATTG
promdNR	5′-CAATGGACAAGTCATTGGAAAACATACTAAGGGATAATCCTG
TmL93AF	5′-CAAGGGCGGAATGAATGCGTTCAAACCTCGTGTGGTTATA
TmL93AR	5′-TATAACCACACGAGGTTTGAACGCATTCATTCCGCCCTTG
TmL93AF94AF	5′-CAAGGGCGGAATGAATGCGGCCAAACCTCGTGTGGTTATA
TmL93AF94AR	5′-TATAACCACACGAGGTTTGGCCGCATTCATTCCGCCCTTG
TmD153AF	5′-GCCCTCCGGGAACATGGTGGTGGCCATCGGTGGAGGAACG
TmD153AR	5′-CGTTCCTCCACCGATGGCCACCACCATGTTCCCGGAGGGC
EcF103AF	5′-CAAGTGCACAGCAACAGCGCTATGCGTCCAAGCCCGCGCG
EcF103AR	5′-CGCGCGGGCTTGGACGCATAGCGCTGTTGCTGTGCACTTG
EcF103AM104AF	5′-CAAGTGCACAGCAACAGCGCTGCGCGTCCAAGCCCGCGCG
EcF103AM104AR	5′-CGCGCGGGCTTGGACGCGCAGCGCTGTTGCTGTGCACTTG
GFPMinDF	5′-GAAGGAGATATACATATGAAAGGCTTCCTCAAACGCTTGTTCCGGGGTTCTCATCATC
GFPMinDR	5′-GATGATGAGAACCCCGGAACAAGCGTTTGAGGAAGCCTTTCATATGTATATCTCCTTC
GFPEcMreB2F	5′-GAAAAAATTTCGTGGCATGTTTGGAGGATCCGAACAGCAATTGAAAAAATTTCGTGGCATGTTT CGGGGTTCTCATCATCATCATC
GFPEcMreB2R	5′-GATGATGATGATGAGAACCCCGAAACATGCCACGAAATTTTTTCAATTGCTGTTCGGATCCTCCA AACATGCCACGAAATTTTTTC
dN209F	5’-GCTTTCAGGATTATCCCTTAGTATGTTTTCCAATGACTTGTCCATTG
dN209R	5’-CAATGGACAAGTCATTGGAAAACATACTAAGGGATAATCCTGAAAGC
pFB209ecoF	5’- CATGATTACGAATTCCCGGGGATCTCG
pFB209HindR	5’- CAATGGACAAGTCATTGGAAAACATACTAAGGGATAATCCTGAAAGC

Supplemental References

Bendezú, F. O., and de Boer, P. A. (2008). Conditional lethality, division defects, membrane involution, and endocytosis in *mre* and *mrd* shape mutants of *Escherichia coli*. J Bacteriol *190*, 1792-1811.

Bendezú, F. O., Hale, C. A., Bernhardt, T. G., and de Boer, P. A. (2009). RodZ (YfgA) is required for proper assembly of the MreB actin cytoskeleton and cell shape in *E. coli*. EMBO J *28*, 193-204.

Miroux, B., and Walker, J. E. (1996). Over-production of proteins in *Escherichia coli:* mutant hosts that allow synthesis of some membrane proteins and globular proteins at high levels. J Mol Biol *260*, 289-298.

van den Ent, F., Amos, L. A., and Löwe, J. (2001). Prokaryotic origin of the actin cytoskeleton. Nature *413*, 39-44.

van den Ent, F., and Löwe, J. (2006). RF cloning: a restriction-free method for inserting target genes into plasmids. J Biochem Biophys Methods 6*7,* 67-74.
