# Supplementary figures and images for: Direct Membrane Binding by Bacterial Actin MreB
Source: Mol Cell. 2011 Aug 5;43(3-6):478–87. doi: 10.1016/j.molcel.2011.07.008 (PMC3163269; doi:10.1016/j.molcel.2011.07.008)

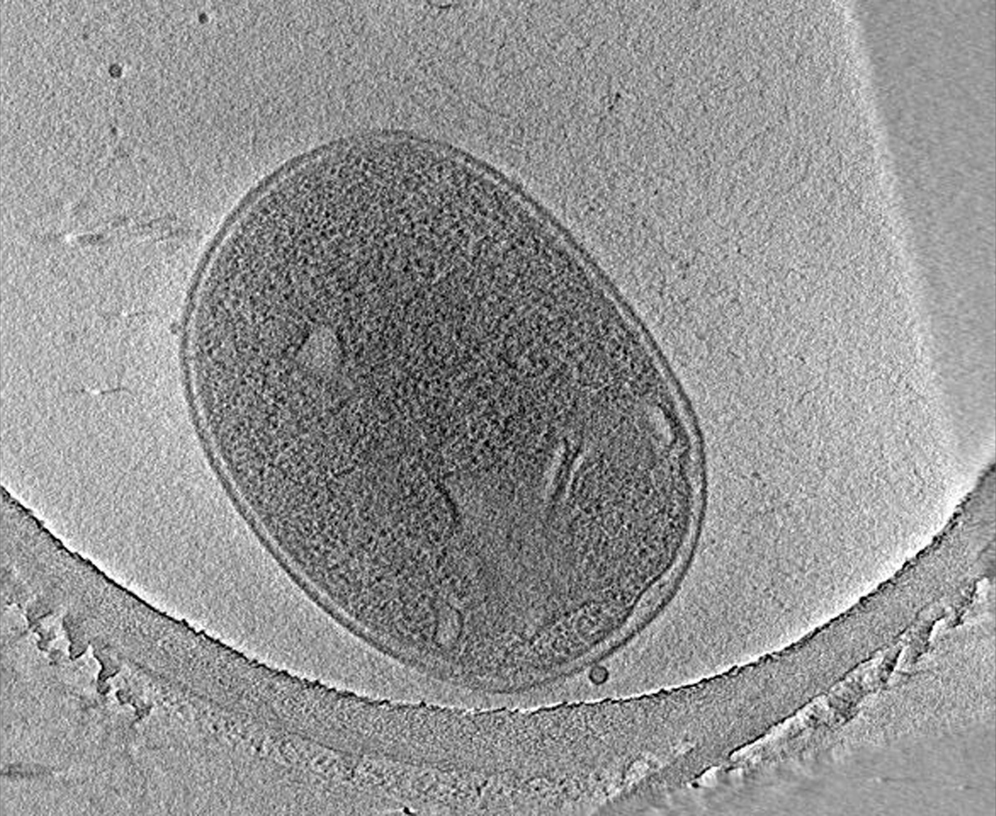

Supplement: Movie S1. 3D Electron Cryotomography Reconstruction of a Rapidly Frozen E. coli Cell, Overexpressing High Levels of TmMreB — TmMreB induces membrane invaginations and curvature, and patches of apparent attachment between membrane surfaces can be observed at the edges of the cell. Related to Figure 1. [file mmc2.jpg]

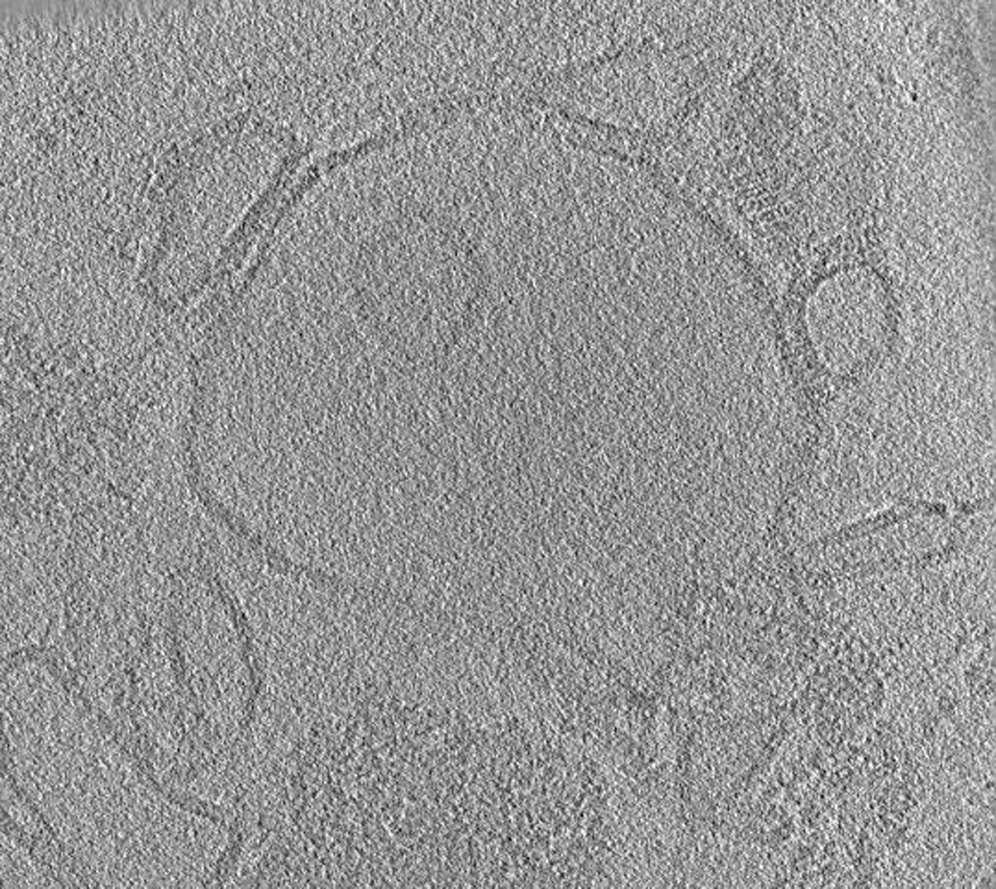

Supplement: Movie S2. 3D Electron Cryotomography Reconstruction of Lipid Vesicles in the Presence of TmMreB and AMP-PNP — Extensive patches of attachment between adjacent membranes can be observed. Double filaments of TmMreB filaments can be resolved, and the double bilayer in an internal, protected liposome is visible. Related to Figure 2. [file mmc3.jpg]
